# Soil bacterial community changes along elevation gradients in karst graben basin of Yunnan-Kweichow Plateau

**DOI:** 10.3389/fmicb.2022.1054667

**Published:** 2022-12-22

**Authors:** Qiang Li, Jiangmei Qiu, Yueming Liang, Gaoyong Lan

**Affiliations:** ^1^Key Laboratory of Karst Ecosystem and Treatment of Rocky Desertification, MNR, Key Laboratory of Karst Dynamics, MNR & GZAR, Institute of Karst Geology, Chinese Academy of Geological Sciences, Guilin, China; ^2^International Research Center on Karst under the Auspices of UNESCO, Guilin, China

**Keywords:** karst graben basin, Yunnan-Kweichow Plateau, elevation gradients, soil bacterial structure patterns, *Bradyrhizobium*

## Abstract

Elevation gradients could provide natural experiments to examine geomorphological influences on biota ecology and evolution, however little is known about microbial community structures with soil depths along altitudinal gradients in karst graben basin of Yunnan-Kweichow Plateau. Here, bulk soil in A layer (0 ~ 10 cm) and B layer (10 ~ 20 cm) from two transect Mounts were analyzed by using high-throughput sequencing coupled with physicochemical analysis. It was found that the top five phyla in A layer were Proteobacteria, Acidobacteria, Actinobacteria, Bacteroidetes, and Verrucomicrobia, and the top five phyla in B layer were Proteobacteria, Acidobacteria, Actinobacteria, Verrucomicrobia, and Chloroflexi in a near-neutral environment. Edaphic parameters were different in two layers along altitudinal gradients. Besides that, soil microbial community compositions varied along altitudinal gradient, and soil organic carbon (SOC) and total nitrogen (TN) increased monotonically with increasing elevation. It was further observed that Shannon indexes with increasing altitudes in two transect Mounts decreased monotonically with significant difference (*p* = 0.001), however beta diversity followed U-trend with significant difference (*p* = 0.001). The low proportions of unique operational taxonomic units (OTUs) appeared at high altitude areas which impact the widely accepted elevation Rapoport’s rules. The dominant *Bradyrhizobium* (alphaproteobacterial OTU 1) identified at high altitudes in two layers constitutes the important group of free-living diazotrophs and could bring fixed N into soils, which simultaneously enhances SOC and TN accumulation at high altitudes (*p* < 0.01). Due to different responses of bacterial community to environmental changes varying with soil depths, altitudinal gradients exerted negative effects on soil bacterial communities *via* soil physical properties and positive effects on soil bacterial diversities *via* soil chemical properties in A layer, however the results in B layer were opposite. Overall, our study is the first attempt to bring a deeper understanding of soil microbial structure patterns along altitudinal gradients at karst graben basin areas.

## Introduction

Complex landforms shaped by nature as an aspect of the combined and interacting influences of geology, climate, time, biota and the secondary composite products of those interactions provide the habitat for life on earth (Phillips, 2016). Moreover, landforms can be distributed following an altitudinal gradient, which result in environmental changes such as temperature and humidity. Consequently, altitudinal gradients provide the most powerful ‘natural experiments’ for examining the hypothesis about geomorphological influences on biota ecology and evolution (Körner, 2007). The research on characteristics, origin and evolution of fauna and flora along altitudinal gradients has long been the focus (Brown, 2001; Sundqvist et al., 2013; Bhat et al., 2020). Based on 441 group data of fauna and flora along altitudinal gradients across Northern and Southern hemispheres, it was found that most elevational diversity curves were skewed positively, that is maximum diversity below the middle of the gradient (Guo et al., 2013). Though soil microorganisms play important roles in material cycles and energy flow in nature, only few studies were did to examine their diversity patterns along altitudinal gradients and soil microbes do not follow elevational diversity patterns of plants and animals (Bryant et al., 2008; Fierer et al., 2011; Keller, 2022).

The distribution patterns of soil microbiome along altitudinal gradients are multiple, complex and changeable. For example, the taxon richness and phylogenetic diversities of soil bacteria decreased monotonically with increasing elevation in Colorado and Southwestern Tibetan Plateau (Bryant et al., 2008; Shen et al., 2019), however Cai et al. (2020) found that soil bacterial and fungal community diversities increased monotonically in northwest Yunnan plateau, China. Moreover, the hump-backed trend in bacterial diversities from Mount Fuji (Singh et al., 2012) as well as declines, increases, mid-elevation or no discernable trend in soil microbial diversities (Looby and Martin, 2020) were found. Moreover, it is a common belief that the number of endemic species decreased and their proportions increased with increasing altitude (Vetaas and Grytnes, 2002; Zhou et al., 2019). However, Grau et al. (2007) found that the proportion of endemic bryophytes with other plant groups in Nepal at the highest altitudes decreased. The multiple microbe patterns along altitudinal gradients may have been due to edaphic factors that shaped the microbial diversities and community compositions varying with the changed sampling areas. Secondly, the responses of soil microorganisms to above-and below-ground ecosystems were out of sync. Consequently, these studies have not uncovered the soil microbial patterns along altitudinal gradients on global scales, especially without representative samples from karst area (Bryant et al., 2008; Singh et al., 2012; Shen et al., 2019; Cai et al., 2020; Looby and Martin, 2020). Therefore, more work is still needed to address soil microbiome patterns along altitudinal gradients to better understand microbial ecology and function (Looby and Martin, 2020).

Despite less reports of soil microbial ecology along karst altitudinal gradients, Hu et al. (2022) and Yan et al. (2022) pointed out the soil microbial diversity, composition and assembly along vegetation succession sequence or calcareous succession process from karst montane areas. Knowing that calcareous soil originating from weathering products of carbonate rocks (limestone, dolomite or marble) has calcium-rich and alkaline characteristics with scales, spatial heterogeneity and temporal dynamics (Yuan, 2001), karst soil microbiome would exhibit different lifestyles and adaptive strategies from non-karst soil. Then, studying their distribution patterns and community characters may reveal the distinctive groups from karst montane areas. Moreover, the response of bacterial community to environmental change varies with surface soil depths (Barbour et al., 2022). Due to greatest number and largest spatial distribution sites with sampling depths from 0 to 10 cm (Wieder et al., 2021) and most karst soil with depths of 0 ~ 20 cm (Yan et al., 2022), the previous studies mainly focused on soil microbial ecology with depths of 0 ~ 10 cm (defined as A layer) or 0 ~ 20 cm. To provide more information on their vertical variability (Qiu et al., 2020) and identify the bacterial community patterns along elevation gradients in karst graben basin of Yunnan-Kweichow Plateau, bulk soil from two layers (A layer and 10 ~ 20 cm defined as B layer) were collected, respectively, for analysis by using high-throughput sequencing coupled with physicochemical analysis. To reduce the knowledge gap, we focus on the following issues in this study: (i) The diversity pattern of soil bacteria in two layers along altitudinal gradients is different. (ii) The proportions of endemic bacteria increase or decrease along altitudinal gradients. (iii) What are the relationships among altitudinal gradients, soil physical/chemical properties, and soil bacterial communities?

## Materials and methods

### Study sites

Eighty soil samples were collected in Xibeile Village from Mengzi City of Yunnan-Kweichow Plateau, China. At this area, karst intermountain basins, namely karst graben basins are typical due to the subsidence and dissolution of fault blocks caused by Cenozoic tectonic uplift. The geomorphic zones are distinct, neotectonic movements are intense, water resource distribution is uneven, soil and vegetation zonings are prominent, vertical climate variations are significant, and regional differences in human activities are large (Wang, Y. et al., 2017). Moreover, red calcareous soil with high Fe_2_O_3_, Al_2_O_3_, and SiO_2_ contents is widely distributed at this area. Due to uneven depths of calcareous soil, 45 soil samples from A layer and 35 soil samples from B layer were collected, respectively. Because intense neotectonic movements resulted in discontinuous elevation, two transects with altitude interval of 505 m between Mount Cuomodi (CMD, altitude from 1,844 to 1,997 m) and Mount Wugongshan (WGS, altitude from 1,290 to 1,339 m) were investigated at this area (Table 1). The plant species at here were *Arundinella setosa*, *Dodonaea viscosa* (L.) Jacq., *Bothriochloa ischaemum* (L.) Keng., *Bidens pilosa* L., *Hedera nepalensis* var. *sinensis* (Tobl.) Rehd., *Parthenium hysterophorus* L., and *Alnus ferdinandi-coburgii* Schneid. The bare rock rates at this area were 2.7–28.9% (Yin et al., 2020). Moreover, the average annual rainfall at this area was 2026.5 mm and the average annual temperature was 16.3°C.

**Table 1:** Environmental parameters with soil depth changes along altitudinal gradients in karst graben basin of Yunnan-Kweichow Plateau.

		Altitude (m)	T (°C)	EC (ms/m)	SM (%)	SOC (g/kg)	TN (g/kg)	TP (g/kg)	AK (g/kg)	pH
(0–10 cm)A layer	CMD1	1997	6.13 ± 0.6d	45.25 ± 6.55f	12.75 ± 2.21d	127.81 ± 45.91a	8.25 ± 3.31a	0.68 ± 0.04c	199.03 ± 55.85c	6.41 ± 0.07c
	CMD2	1966	5.18 ± 0.43e	60 ± 3.56e	17.6 ± 1.72d	91.32 ± 65.08b	7.33 ± 5.04ab	0.77 ± 0.23bc	122.35 ± 51.06 cd	6.4 ± 0.22c
	CMD3	1947	5.43 ± 0.76ed	73.5 ± 4.12d	32.6 ± 2.87bc	17.28 ± 3.75c	1.62 ± 0.25c	0.3 ± 0.08d	68 ± 19.26d	6.73 ± 0.23ab
	CMD4	1934	7.5 ± 0.79c	70.5 ± 1.73de	34.78 ± 2.2b	20.05 ± 0.83c	1.83 ± 0.1c	0.61 ± 0.22c	55 ± 6.55d	6.55 ± 0.09bc
	CMD5	1873	6.06 ± 0.77d	96.8 ± 6.3c	45.1 ± 8.08a	38.21 ± 13.41c	3.43 ± 1.17bc	0.92 ± 0.06b	265.18 ± 53.73bc	6.65 ± 0.07b
	CMD6	1844	6.88 ± 0.35 cd	96.75 ± 7.59c	48.33 ± 1.36a	20.86 ± 0.65c	1.71 ± 0.03c	0.71 ± 0.02c	352.75 ± 48.2b	6.6 ± 0.19bc
	WGS1	1,339	12.65 ± 0.17b	112.5 ± 5.2bc	22.45 ± 6.22 cd	62.78 ± 3.62bc	4.87 ± 0.48b	0.83 ± 0.08bc	169.08 ± 84.08 cd	6.66 ± 0.24ab
	WGS2	1,324	14.5 ± 0.44a	111.25 ± 8.5bc	25.48 ± 10.19c	48.84 ± 17.88c	3.07 ± 0.32bc	0.67 ± 0.07c	193.5 ± 12.04c	6.68 ± 0.04ab
	WGS3	1,312	13.33 ± 0.46b	101.25 ± 4.03c	14.48 ± 5.02d	45.67 ± 12.21c	3.89 ± 1.35bc	0.68 ± 0.12c	279.55 ± 178.92bc	6.6 ± 0.18bc
	WGS4	1,299	13.88 ± 0.5ab	120.75 ± 5.25b	34.85 ± 2.28b	17.73 ± 1.39c	1.51 ± 0.12c	0.64 ± 0.03c	127.38 ± 10.74 cd	6.86 ± 0.07a
	WGS5	1,290	12.6 ± 0.27b	153.25 ± 20.68a	24.95 ± 0.82c	24.34 ± 1.29c	1.85 ± 0.27c	1.17 ± 0.31a	574.25 ± 140.62a	6.32 ± 0.09c
(10–20 cm)B layer	CMD1	1997	8.5 ± 1.83e	56.67 ± 5.86c	21.67 ± 7.44c	112.82 ± 59.74a	7.6 ± 4.29a	0.51 ± 0.07c	80.13 ± 8.64bc	6.55 ± 0.26ab
	CMD2	1966	8.1 ± 0.71e	73.5 ± 17.68c	23.35 ± 12.09c	31.05 ± 6.13bc	2.35 ± 0.2bc	0.55 ± 0.01c	42.3 ± 16.12bc	6.21 ± 0.1b
	CMD3	1947	8.93 ± 0.36de	73 ± 4.08c	48.2 ± 4.21ab	9.9 ± 6.56c	1.07 ± 0.47c	0.22 ± 0.02d	30.95 ± 10.75c	6.58 ± 0.08ab
	CMD4	1934	9.63 ± 0.31d	67.5 ± 6.35c	47.48 ± 3.18ab	11.26 ± 0.86c	1.08 ± 0.19c	0.51 ± 0.02c	31.75 ± 10.98c	6.66 ± 0.19a
	CMD5	1873	8.3 ± 0.62e	109.6 ± 9.45b	52.32 ± 4.8a	23.24 ± 8.92c	2.1 ± 0.61bc	0.82 ± 0.09b	119.72 ± 28.13b	6.8 ± 0.12a
	CMD6	1844	8.43 ± 0.55e	97.75 ± 3.59b	51.88 ± 1.96a	12.4 ± 0.27c	1.07 ± 0.04c	0.57 ± 0.03c	126.08 ± 13.69b	6.57 ± 0.22ab
	WGS1	1,339	14.87 ± 0.15b	106.33 ± 4.16b	21.63 ± 6.19c	52.33 ± 5.4b	3.49 ± 0.73b	0.72 ± 0.1bc	140.07 ± 22.98b	6.63 ± 0.05ab
	WGS2	1,324	16.55 ± 0.07a	111.5 ± 0.71b	36 ± 0bc	33.66 ± 0.6bc	2.05 ± 0.04bc	0.61 ± 0c	129.5 ± 22.77b	6.64 ± 0.09ab
	WGS4	1,299	15.73 ± 0.13ab	116.5 ± 15.46b	42.13 ± 1.56b	9.11 ± 2.35c	0.84 ± 0.21c	0.52 ± 0.02c	73.95 ± 6.76bc	6.62 ± 0.27ab
	WGS5	1,290	13.45 ± 0.26c	168.5 ± 30.73a	28.78 ± 3.2c	21.75 ± 1.25c	1.55 ± 0.19bc	0.96 ± 0.18a	370.78 ± 169.2a	6.41 ± 0.14b

Different lower case letters represent significant difference from different sampling sites in the same soil layer (*p <* 0.05). Data are means ± standard error. Five duplicates in CMD5 and four repetitions in other sample sites from A layer. Three duplicates in WGS1, WGS2, CMD1, and CMD2, no samples in WGS3, and the same repetitions in other sample sites from B layer as in A layer.

### Soil sample collection

Six sampling sites in Mount CMD and 5 sampling sites in Mount WGS were investigated in January 2018. At each randomly selected sampling site almost without man-made management to avoid pseudo-replication, at least three replicates of soil samples with 5 m distances in every soil layer were randomly collected along S-shapes. If soil layer was less than 20 cm, only soil samples in A layer were collected. If soil layer was more than 20 cm, soil samples in two layers were simultaneously collected. After substance invasion removed, the soil samples were evenly divided into two parts and kept in sterile polyethylene bags for future work. One part was used for edaphic analysis and another part was kept in −80°C for soil bacterial community analysis. The detailed sampling information is listed in Supplementary Table S1.

### Edaphic analysis

Soil organic carbon (SOC) and total nitrogen (TN) were determined on SerCon Integra 2 Elemental Analyzer (Sercon Ltd., England). Soil pH, total phosphorus (TP) and available potassium (AK) were determined according to the methods described in Hu et al. (2022) and Yan et al. (2022). Soil temperature (T), soil moisture (SM), and electrical conductivity (EC) were measured *in situ* by soil three-parameter tachometer (UK three-parameter tachometer HH2/WET).

### Sequencing of 16S rRNA genes and bioinformatic analysis

Soil DNA extraction and high-throughput sequencing were performed in Magigene Ltd., China. Their detailed protocols were described in Hu et al. (2022) and Yan et al. (2022). The V3-V4 region of 16S rRNA gene was amplified using PCR primers of 338F and 806R (Wang, J. et al., 2017) and sequenced on the Illumina HiSeq 2,500 platform (Illumina Inc., San Diego, CA, United States). The sequence reads were deposited in the NCBI Sequence Read Archive under the accession number PRJNA514872.

Raw sequencing reads were processed on QIIME 1.9.1. About 4,637,609 raw reads of 80 soil samples filtered with length less than 300 bp or average base quality score more than 20 resulted in 1,446,651 high-quality and chimera-free reads, with a minimum sequencing depth of 8,138 reads per sample, according to Silva v.123 16S rRNA database. All clean sequences were grouped into operational taxonomic units (OTUs) based on a genetic similarity of 97%. Alpha diversities (Chao 1, Simpson, Shannon, observed OTUs, PD whole tree and Goods coverage) and beta diversity based on Bray-Curtis metrics were calculated based on rarefied OTU tables. The Goods coverage of all samples was more than 93% indicating that the achieved sequencing depth was sufficient for sequential studies. The detailed data are shown in Supplementary Table S1.

### Statistical analysis

One-way ANOVA and Pearson’s correlation analyses (two-tailed test) were carried out with SPSS 19.0. to perform statistical analysis. The Origin 8.5 software was used to illustrate the variations of mean relative abundances at phylum level and the alpha diversities, as well as the influence of altitudinal gradients on Shannon index.

Moreover, principal coordinate analysis (PCoA) was used to assess the influence of altitudinal gradients on the similarity/dissimilarity of soil bacterial communities by using beta diversity data based on Bray-Curtis metrics. Redundancy analysis (RDA) was carried out by Canoco 5 software to display the relationships between environmental factors and identified phyla in our study. The heat map was performed by using R studio 2.15.1 to detect the relationships between the most abundant OTUs and environmental factors, as well as the relative frequency of the most abundant OTUs in each sampling site. To explore the influence of altitudinal gradients on the proportions of shared and unique OTUs, the OTUs with more than five sequences were used to sort the shared and unique OTUs at different altitudes for network-based visualization generated with Cytoscape 3.6.1 (Shen et al., 2019). The partial least squares path model (PLS-PM) and PASSaGE 2 software was used to detect the relationships between altitudinal gradients, soil physical/chemical properties, soil bacterial communities based on the most abundant OTUs, and soil bacterial alpha diversities. Partial Mantel test was carried to recognize the influence of altitudinal gradients and soil physical/chemical properties on soil bacterial communities. Moreover, analysis of similarity (ANOSIM) test was performed with the vegan R package to determine the statistical differences of beta diversities along altitudinal gradients (Anderson and Walsh, 2013).

## Results

### Edaphic parameters along altitudinal gradients

In general, the monotonically increased SOC and TN had no obvious changes (Table 1). In our study, soil pH was in a near-neutral environment. T, SM and EC in A layer were lower than those in B layer, however TP, AK, SOC and TN in A layer were higher than those in B layer. Besides that, altitudinal gradients had significantly negative effects on T, EC and AK in different soil layers (*p* < 0.05), and had significantly negative and positive effects on TP and SM, respectively, in B layer (*p* < 0.05, Supplementary Table S2). Despite all this, the influence of altitudinal gradients on edaphic parameters in the two transects was different. Altitudinal gradients had significantly positive effects on TN and SOC in A layer from Mount WGS (*p* < 0.05), and in the two layers from Mount CMD (*p* < 0.05), and had significantly negative effects on pH in A layer from Mount CMD (*p* < 0.05).

### Soil microbial community structures and diversities along altitudinal gradients

**Figure 1:**
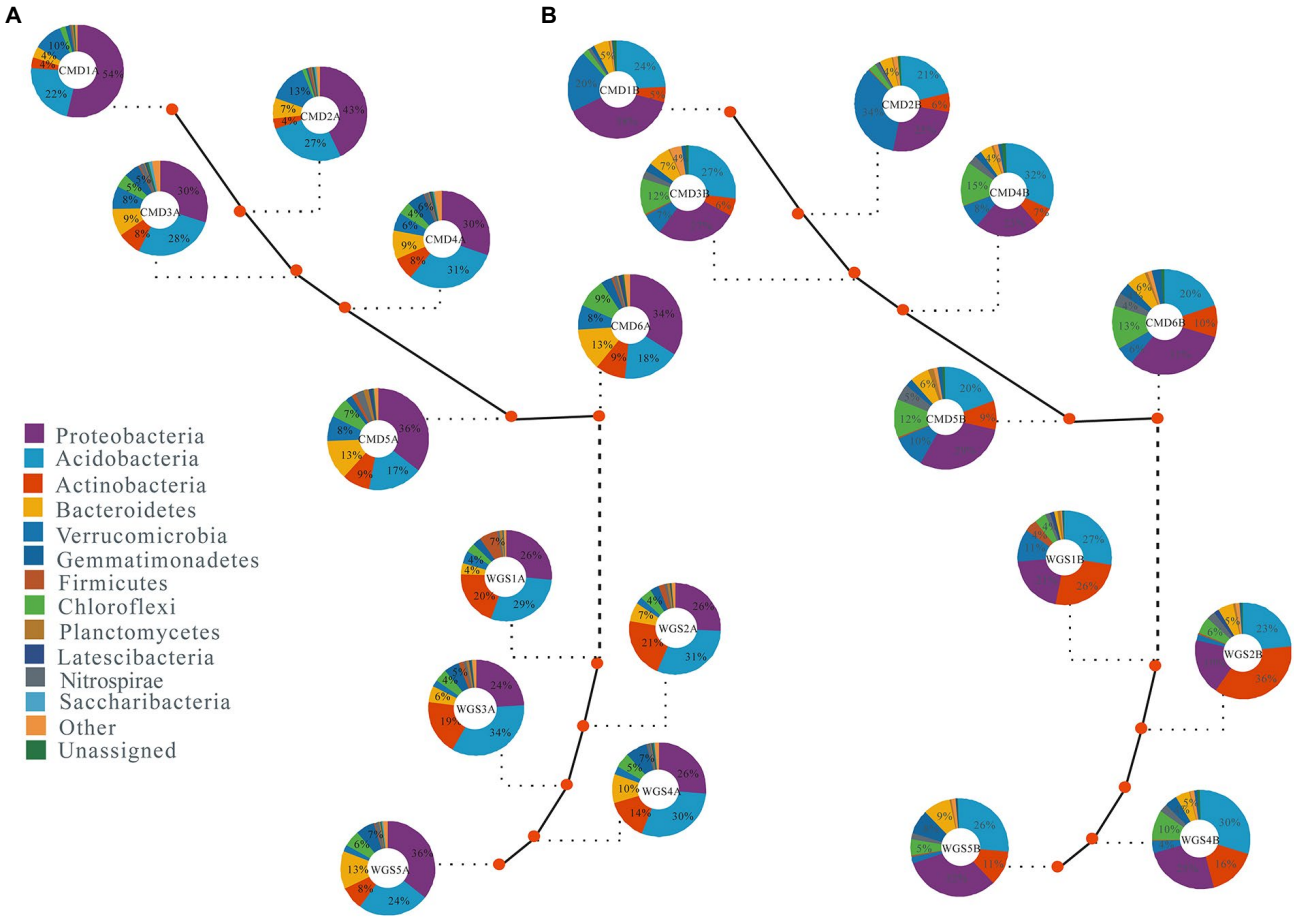
The mean relative abundance (≥0.1%) of dominant soil bacterial phyla in karst graben basin of Yunnan-Kweichow Plateau **(A)** indicating A layer and **(B)** indicating B layer. The mean relative abundance less than 0.1% were classified into other.

Of all the reads, ≥94.7% were assigned to 10 phyla (e.g., Proteobacteria, Acidobacteria, Actinobacteria, Firmicutes, Bacteroidetes, Gemmatimonadetes, Chloroflexi, Verrucomicrobia, Planctomycetes, and Nitrospirae) in A layer, and ≥ 95.1% were assigned to 11 phyla (e.g., Acidobacteria, Actinobacteria, Proteobacteria, Verrucomicrobia, Firmicutes, Chloroflexi, Gemmatimonadetes, Bacteroidetes, Planctomycetes, Nitrospirae, and Latescibacteria) in B layer. Moreover, their abundances in the two layers along altitudinal gradients were different (Figure 1). The top five phyla with mean relative abundances in A layer were Proteobacteria (24% ~ 43%), Acidobacteria (17% ~ 34%), Actinobacteria (4% ~ 21%), Bacteroidetes (4% ~ 13%) and Verrucomicrobia (2% ~ 13%), and the top five phyla in B layer were Proteobacteria (19% ~ 38%), Acidobacteria (20% ~ 32%), Actinobacteria (5% ~ 36%), Verrucomicrobia (2% ~ 34%), and Chloroflexi (2% ~ 15%). The mean relative abundances of Proteobacteria followed U-shaped patterns, Actinobacteria, Firmicutes, Bacteroidetes, Chloroflexi, Planctomycetes, and Nitrospirae had hump-shaped patterns, Gemmatimonadetes decreased monotonically while Verrucomicrobia and Acidobacteria increased monotonically with increasing altitudes in A layer (Supplementary Figure S1). By contrast, the mean relative abundances of Acidobacteria, Proteobacteria, and Bacteroidetes followed U-shaped patterns, Firmicutes, Chloroflexi, Planctomycetes, Nitrospirae, Latescibacteria, and Actinobacteria had hump-shaped patterns, Verrucomicrobia increased monotonically as well as Gemmatimonadetes decreased monotonically with increasing altitudes in B layer.

**Figure 2:**
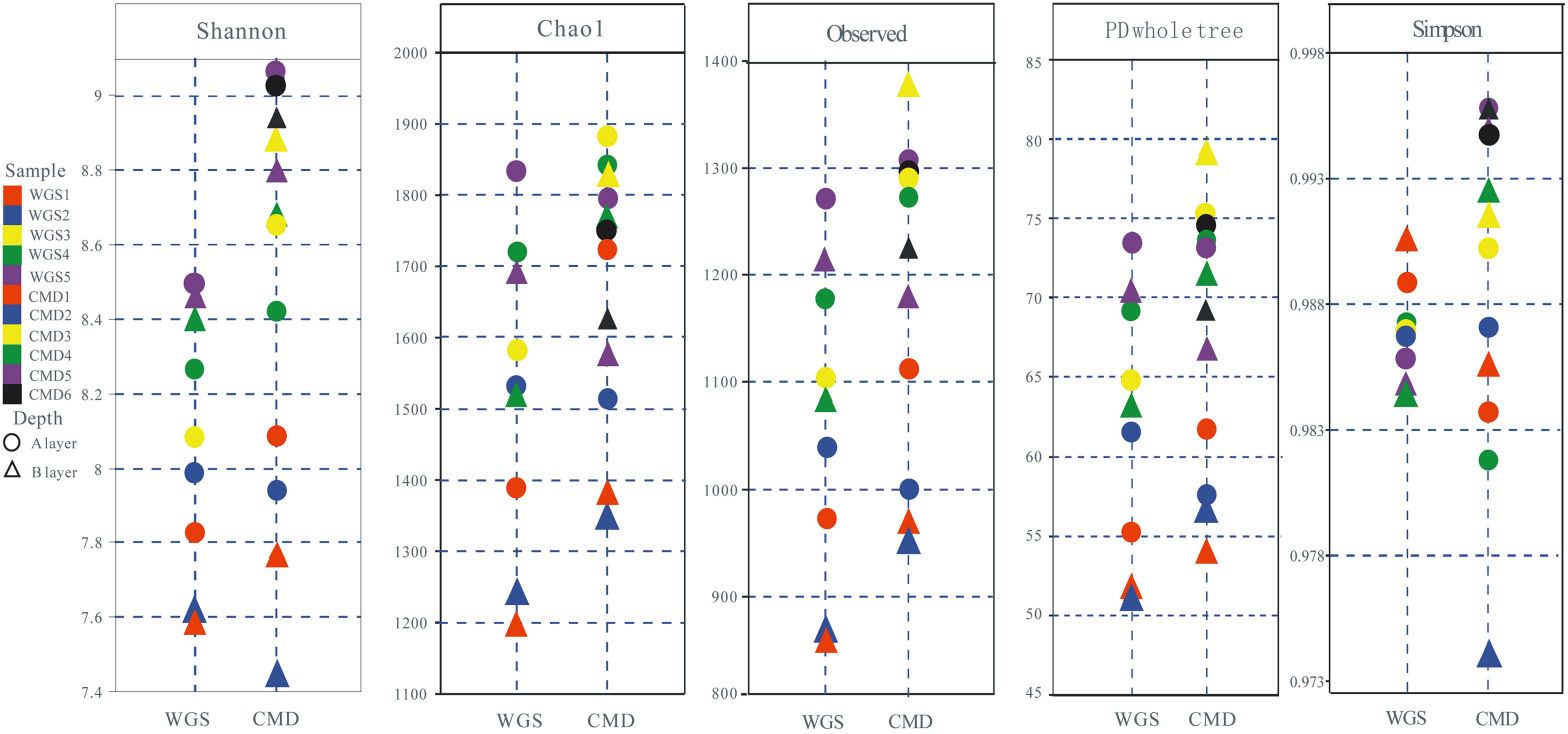
Alpha diversity of soil bacteria from Mount WGS and Mount CMD in karst graben basin of Yunnan-Kweichow Plateau.

**Figure 3:**
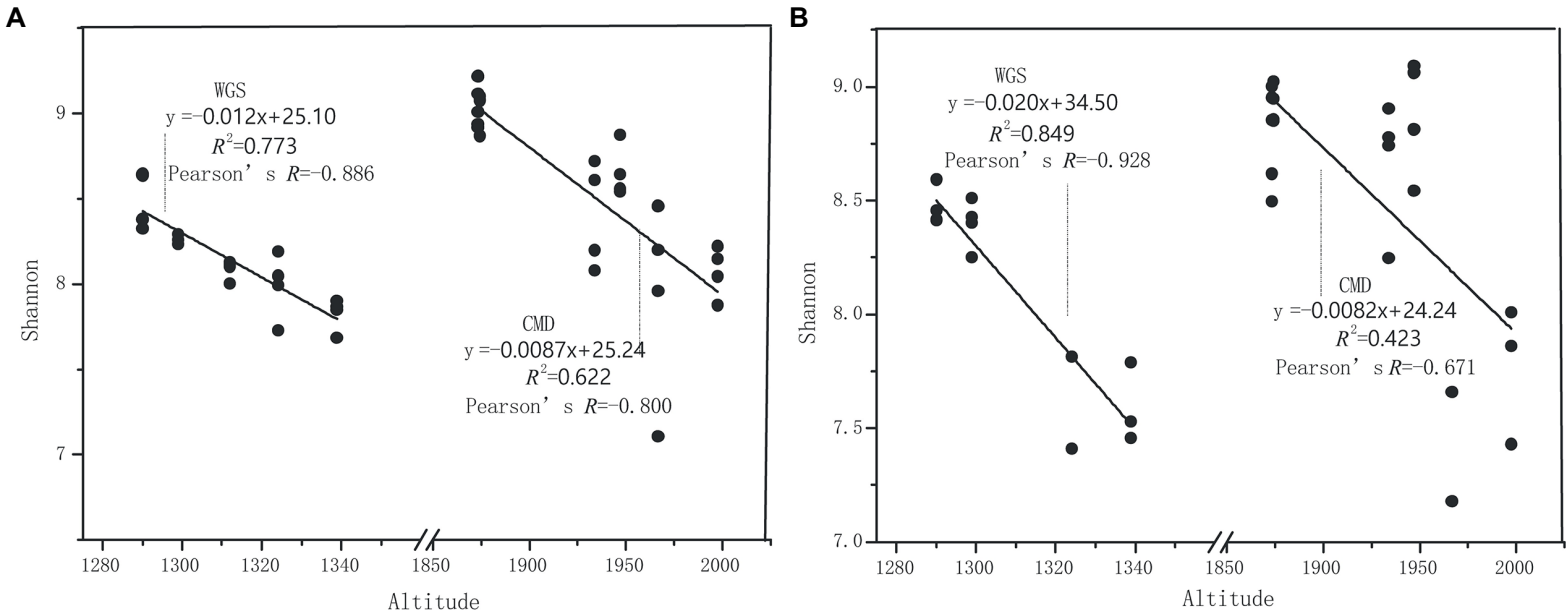
The relationships between altitudinal gradients and Shannon indexes in karst graben basin of Yunnan-Kweichow Plateau **(A)** indicating A layer and **(B)** indicating B layer.

**Figure 4:**
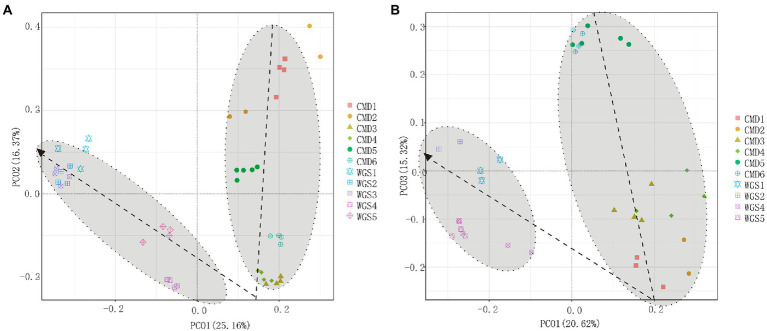
PCoA plots indicating influences of altitudinal gradients on the similarity/dissimilarity of soil bacterial communities by using beta diversity data based on Bray-Curtis metrics **(A)** indicating A layer and **(B)** indicating B layer.

The alpha diversities along altitudinal gradients were higher in Mount CMD than those in Mount WGS, and were also higher in A layer than those in B layer (Figure 2). Considering that Shannon diversity usually was recommended to analyze microbial diversity, it was selected as the metric to investigate the effects of altitudinal gradients on alpha diversities. It was found that Shannon indexes decreased monotonically with increased elevation in Mount CMD and Mount WGS (Figure 3). Moreover, similar variations were also found in the two layers. The similarities and differences in soil bacterial community structures can be described by using beta diversity based on Bray-Curtis distances. The results indicated that two independent bacteria groups from A layer and B layer were identified in Mount CMD and Mount WGS, and U-trend was formed, though no monotonic changes appeared with increasing altitude (Figure 4). Moreover, ANOSIM revealed that altitudinal gradients exerted significantly influences on soil bacteria diversities (Alpha diversities—*R* = 0.399, *P* = 0.001 in A layer, alpha diversities—*R* = 0.597, *P* = 0.001 in B layer, beta diversity based on Bray-Curtis—*R* = 0.862, *P* = 0.001 in A layer, and beta diversity based on Bray-Curtis—*R* = 0.985, *P* = 0.001 in B layer), as seen in Supplementary Figure S2.

**Figure 5:**
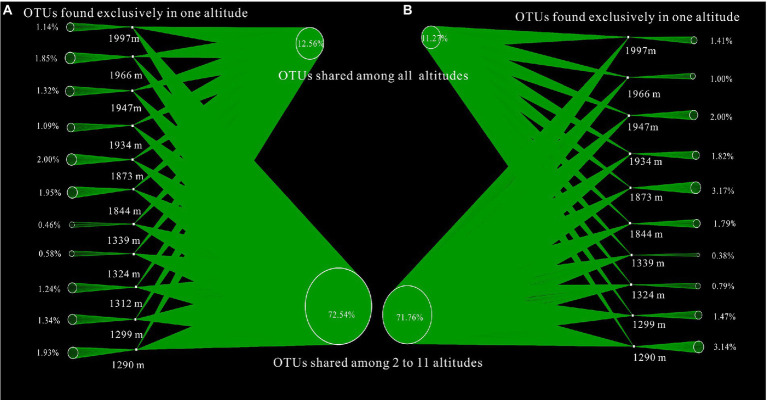
Influences of altitudinal gradients on the proportions of shared and unique OTUs based on network-based visualization **(A)** indicating A layer and **(B)** indicating B layer.

To better evaluate the similarity and difference of bacterial communities along altitudinal gradients, network-based visualization with the proportions of shared and unique OTUs was applied. The proportions of shared OTUs in A layer and B layer were 12.56% (shared OTUs = 496, total OTUs = 3,948) and 11.27% (shared OTUs = 383, total OTUs = 3,407) respectively (Figure 5). The proportions of unique OTUs with increasing altitude exhibited the similar variation trend to Shannon diversities in Mount CMD and Mount WGS. Moreover, the proportions of unique OTUs in Mount CMD were lower than those in Mount WGS. In any case, the low proportion value of unique OTUs appeared in the joint area of Mount CMD and Mount WGS.

**Figure 6:**
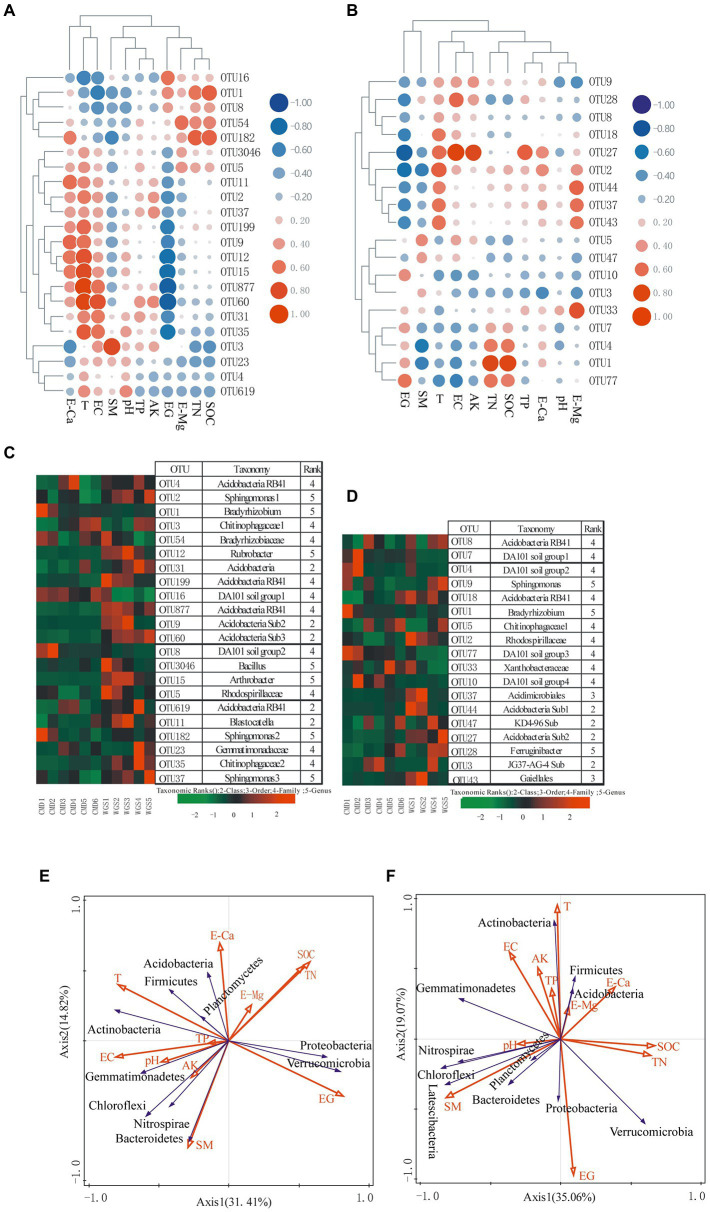
Heat map representing relationships between the most abundant OTUs (>0.5%) and environmental factors **(A)** from A layer and **(B)** from B layer, as well as the relative frequency of the most abundant OTUs (>0.5%) in each sampling site **(C)** from A layer and **(D)** from B layer. RDA plots displaying the relationships between environmental factors and the identified phyla **(E)** from A layer and **(F)** from B layer. EG indicating altitudinal gradient.

Among the most frequent OTUs, only *Rubrobacter* (actinobacterial OTU 12), *Arthrobacter* (actinobacterial OTU 15), *Bradyrhizobium* (alphaproteobacterial OTU 1), and *Sphingomonas* (alphaproteobacterial OTUs 2, 37 and 182) were classified at the genus level in A layer (Figures 6C,D). In contrast, only *Bradyrhizobium* (alphaproteobacterial OTU 1), *Sphingomonas* (alphaproteobacterial OTU 9), and *Ferruginibacter* (Bacteroidetes OTU 28) were classified at the genus level in B layer. The altitudinal gradients had obvious effects on them (Figures 6A,B). In A layer, Actinobacteria-related OTUs (12 and 15) were dominant at low altitudes, and Proteobacteria-related OTUs (1 and 182) were dominant at high altitudes. In contrast, only Proteobacteria-related OTU1 was dominant at high altitudes in B layer.

### Relationships between soil microbial communities and environmental parameters

Heatmap was drawn to reveal relationships between environmental parameters and the most frequent OTUs as well as dominant phyla in this study (Figure 6). In general, only SM showed significantly negative correlations with some OTU clusters in the two layers (*p* < 0.05), and other environmental factors had significantly negative or positive correlations with some OTU clusters (*p* < 0.05, Supplementary Table S3). Moreover, altitudinal gradients, T, EC, SM, SOC, TN, TP, AK and pH had significantly negative or positive correlations with some dominant phyla (*p* < 0.05, Figures 6E,F; Supplementary Table S4).

**Figure 7:**
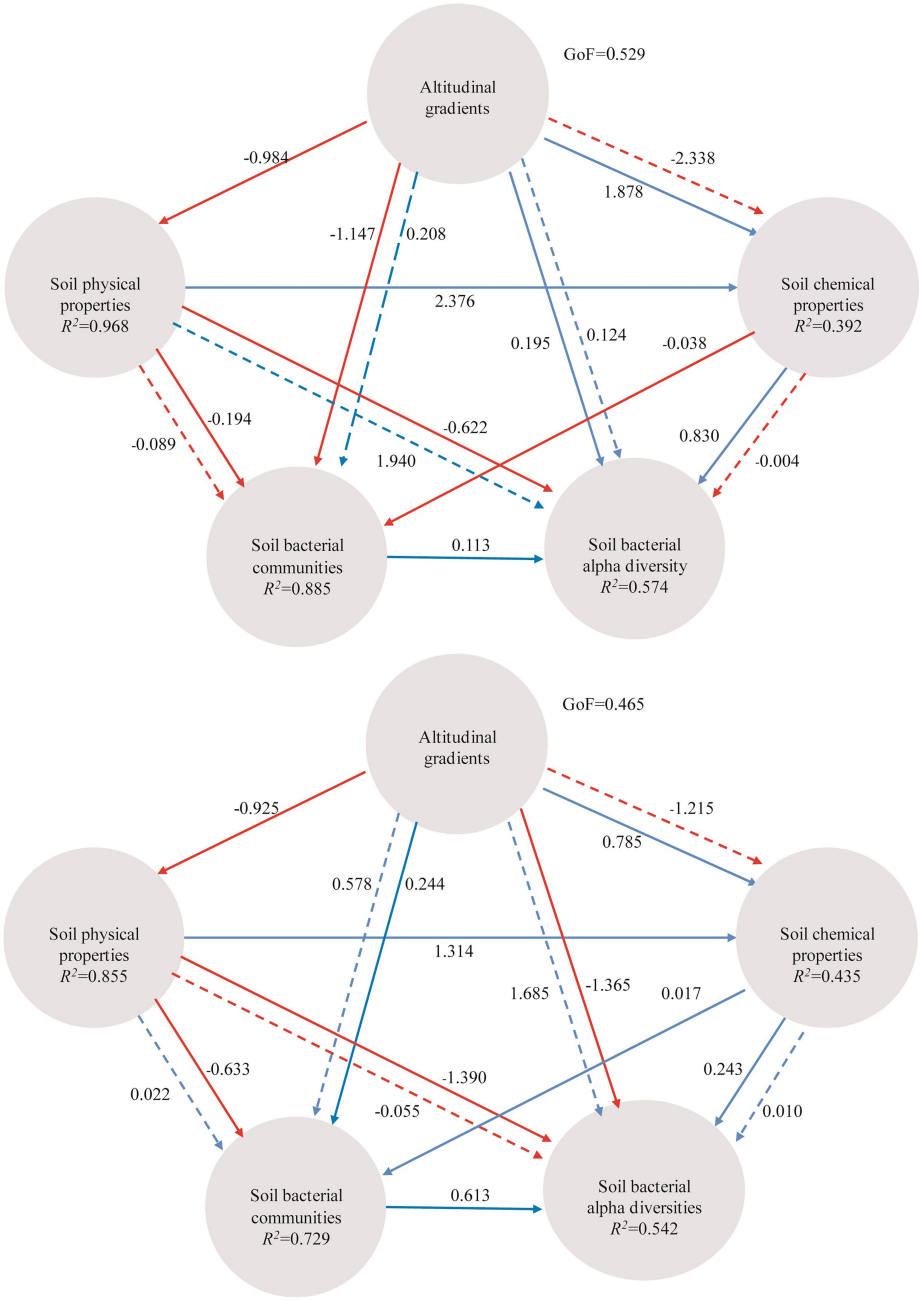
PLS-PM indicating the relationships between altitudinal gradients, soil physical properties (T, EC, and SM), chemical properties (SOC, TP, AK, pH, and TN), soil bacterial communities based on the most abundant OTUs and soil bacterial alpha diversities (Shannon, Simpson and observed OTUs). The path coefficients and the explained variability (*R*^2^) are calculated after 999 bootstraps and reflected in the arrows with blue and red indicating positive and negative effects, respectively (solid line represents direct effect and dotted line represents indirect effect). Models with different structures were assessed using the Goodness of Fit (GoF) statistic, a measure of the overall prediction performance.

Instead of focusing on the relationships between individual environmental factors and soil bacterial communities, partial Mantel test was used to reveal the inter-relationships among altitudinal gradients, soil physical/chemical properties and soil bacterial communities (Table 2). Altitudinal gradients had significant influence on microbial communities from A layer and B layer (*p* < 0.01). Moreover, PLS-PM indicated that altitudinal gradients exerted negative effects on soil physical properties, and exerted positive effects on soil chemical properties *via* soil physical properties (Figure 7). Altitudinal gradients exerted negative effects on soil bacterial communities *via* soil physical properties and positive effects on soil bacterial diversities *via* soil chemical properties in A layer, however the results in B layer were opposite.

**Table 2:** Influence of altitudinal gradients and soil physical/chemical properties on soil bacterial communities by partial Mantel test with soil depth changes in karst graben basin of Yunnan-Kweichow Plateau.

	Effect of	a	a	a	a	b	b	b	b
A layer	Controlling for		b	c	bc		a	c	ac
	Bacterial communities	*r*	*r*	*r*	*r*	*r*	*r*	*r*	*r*
		0.642	0.263	0.638	0.266	0.643	0.266	0.633	0.245
B layer	Effect of	a	a	b	b	b	b		
	Controlling for		c		a	c	ac		
	Bacterial communities	*r*	*r*	*r*	*r*	*r*	*r*		
		0.418	0.408	0.517	0.336	0.5	0.318		

All *p* values are less than 0.01. a. Altitudinal gradients; b. Soil physical properties (T, EC and SM); c. Soil chemical properties (SOC, TP, AK, pH, and TN).

## Discussion

This study showed the results of bacterial community characters, and the relationships between them and environmental factors along altitudinal gradients in karst graben basin of Yunnan-Kweichow Plateau.

### Influence of altitudinal gradients on edaphic parameters

As previous report, climate, topography, and parent material are natural factors affecting SOC storage and distribution (Baveye et al., 2020). The maximum value of SOC was observed at high altitude which also may be due to the low microbial activities or the contributions of endemic microorganism at this area. Soil microorganisms performing SOC cycles involve two main stages: (i) *ex vivo* modification of organics relating to extracellular enzymes, and (ii) *in vivo* turnover of substances controlled by soil microorganisms (Liang et al., 2017). Then, the decreased activities of specific enzymes *via* microbial secretion at high altitude may contribute to high SOC accumulation in karst graben basin of Yunnan-Kweichow Plateau (Kumar et al., 2019), though we did not assess the exoenzyme activities. The contribution of endemic microorganism to SOC accumulation at high altitude will be discussed later in this article. It was well known that 96–98% soil N existed as complex and insoluble polymers which can be broken down by specific soil enzymes produced by specific soil microorganisms (Van Der Heijden et al., 2008). Coupled with the significant correlation between SOC and TN (*p* < 0.01, Supplementary Table S2), high soil TN was found at high altitude.

Moreover, the environmental features at high altitude are typical of low temperature and arid (Li et al., 2019). Consequently, SM and T decreased with increasing altitude. As to EC, it was usually used to indicate soil salt content or chemical supply which decreased with increasing altitudes due to weathering product from carbonate rocks easily transported and carried away by runoff. This phenomenon also occurred in TP and AK, which may be relating to soil drying and re-wetting significantly affecting P and K leached from soils (Gao et al., 2020; Amin et al., 2021). Except for the lower SM and T in A layer reflecting the direct influence of climate change (Kardol et al., 2010), the decreased SOC, TN, TP, AK and EC in deep soil correlates to previous studies (Pham et al., 2018). The high input of plant residues and plant activity could explain their higher levels in top soils (Jobbágy and Jackson, 2001; Pham et al., 2018).

### Influence of altitudinal gradients on soil bacterial communities

Considering that environmental conditions determine soil bacterial compositions (Xun et al., 2015), and the occurrence and functioning of soil bacteria depend on their niches (Banerjee et al., 2018), soil bacterial abundances (e.g., Acidobacteria, Actinobacteria, Proteobacteria, Verrucomicrobia, Firmicutes, Chloroflexi, Gemmatimonadetes, Bacteroidetes, Planctomycetes, Nitrospirae, and Latescibacteria) can follow hump-shaped, decreasing, increasing or U-shaped patterns along altitudinal gradients. These bacteria followed the above patterns due to them at their own optimum conditions though the responses of their ecological lifestyles to altitudinal gradients remain unclear (Nottingham et al., 2018; Dai et al., 2021). Besides that, the different distribution patterns of bacteria taxa in two layers may be due to their response depending on soil depth (Barbour et al., 2022).

The monotonically decreased Shannon indexes with elevation increasing in Mount CMD and Mount WGS indicate that inducible mutations in easily accessed low-altitude areas may promote the enormous appearance of unique/endemic species, especially rare bacterial species (Fitzgerald and Rosenberg, 2019). By contrast, in the harsh environments at high altitudes, only few rare taxa adapting to this environment can be induced. Though unique/endemic species restricted in specific habitats are important contributors to soil microbial diversities (Lynch and Neufeld, 2015), our result was contrary to previous reports that endemic species decreased with elevation increasing (Zhou et al., 2019). Second, and more speculatively, red calcareous soil with spatial heterogeneity and temporal dynamics in Yunnan-Kweichow Plateau may drive the occurrence of high Shannon indexes at low altitude areas because rare taxa acting as ‘seed bank’ could become dominant under proper conditions (Yuan, 2001; Lennon and Jones, 2011; Jiao and Lu, 2020). These reasons also were applied to the variations of other alpha diversities. Moreover, based on independent-monotonically decreasing Shannon indexes from Mount CMD and Mount WGS, it can be speculated that the coordinated patterns of Shannon indexes may be monotonically decreased with elevation increasing though karst graben basin caused sampling discontinuous. Considering that nutrients (e.g., SOC and TN) in surface soils are usually high than those in sub-surface soils (Hayat et al., 2021), and high nutrient accessibilities could cause the imbalances of soil microbial communities and the appearance of atypical nutrient substrates favoring unique bacterial strains (Leeming et al., 2019; Mirmohamadsadeghi et al., 2021), the alpha diversities were higher in A layer than those in B layer. As to higher alpha diversities in Mount CMD than those in Mount WGS, perhaps spatiotemporal heterogeneity at high altitude areas may be the major drivers of microorganism alpha diversity (Banerjee et al., 2018). Though vegetation type has been reported to influence soil bacterial community diversity and composition (Karimi et al., 2018), alpha diversities in our study has no obvious changes with vegetation type variations (Supplementary Figure S3).

It is should be noted that alpha diversity was used as a measure of species richness and beta diversity was used to indicate the compositional dissimilarity at community levels. Though alpha diversities (e. g. Shannon indexes) may be monotonically decreased with elevation increasing, the U-trend of beta diversity in our study was in accordance with previous studies (Nottingham et al., 2018). These results suggested that though the dissimilarity of soil bacterial communities was affected by multiple environmental factors (Dai et al., 2021), changeable conditions at low or high elevation area sustained higher biodiversity than that at long-term stability environment in middle elevation area (Simpson, 1980). Moreover, if continuous samplings were obtained in karst graben basin of Yunnan-Kweichow Plateau, the real changing rules may be not U-trend. Then, beta diversity was the suitable indicator of karst soil bacterial structure patterns along altitudinal gradients in Yunnan-Kweichow Plateau.

Though it is a common belief that endemic species decreased and their proportions increased with elevation increasing (Zhou et al., 2019), the monotonically decreased proportions of unique OTUs with elevation increasing in Mount CMD and Mount WGS might impact the widely accepted opinions, namely elevation Rapoport’s rules (Stevens, 1992). The reason is that soil microbial communities usually contain a large number of low-abundance species (usually referred as the unique/endemic species) and a small number of high-abundance species at low altitude areas, and only few rare taxa at high altitude areas can be induced (Lennon and Jones, 2011; Jiao and Lu, 2020). In this respect, high proportions of unique OTUs appear at low altitude areas, and low proportions of unique OTUs appear at high altitude areas. This result conforms to the changed regularities of Shannon indexes. Moreover, due to high proportions of unique OTUs appearing at low altitude areas, the proportions of unique OTUs in Mount CMD were lower than those in Mount WGS, which in general followed hump-shaped patterns along altitudinal gradients perhaps due to the discontinuous ridge at his area. In spite of this, their ecology mechanisms which are contrary to elevation Rapoport’s rules in karst graben basin of Yunnan-Kweichow Plateau are not well clear and further research is needed.

### The interactions between environmental factors and soil bacterial communities along altitudinal gradients

At phylum level, the associations between environmental factors and soil bacterial taxa varied, which may have been due to environmental filtering. That is, specific environmental factors favor the formation of particular soil bacterial communities (Delgado-Baquerizo et al., 2018; Nottingham et al., 2018). In this respect, a certain environmental factor may promote the fast-growth of some bacteria but could restrict the growth of other bacteria (Delgado-Baquerizo et al., 2018; Langenheder and Lindström, 2019). Consequently, the most frequent OTUs, especially those classified at the genus level had the significantly negative or positive correlations with environmental factors in our study (*p* < 0.05). Significantly, *Bradyrhizobium* (alphaproteobacterial OTU 1) appearing in all layers had significantly positive relationships with SOC and TN (*p* < 0.01), which may have been due to them performing free-living N_2_ fixation (Tao et al., 2021). Tao et al. (2021) also have reported that *Bradyrhizobium* could constitute the important group of free-living diazotrophs and potentially bring a amount of fixed N into soils, which simultaneously enhances SOC accumulation. Moreover, *Bradyrhizobium* could be tolerant to stressors due to them harboring *HspQ* gene which encodes a chaperone protein to combat detrimental effects (Shimuta et al., 2004; Tao et al., 2021). Consequently, Proteobacteria-related OTU1 was dominant at high altitudes in all layers.

Though a particular factor can be examined by experiments to obtain their role in microbial ecology, the studies about soils reacting to multifactor changes at a time are less (Rillig et al., 2019). In fact, soils are usually influenced by multiple-factors due to their spatial heterogeneity and temporal dynamics (Baldrian, 2019; Rillig et al., 2019). Understanding the impacts of multiple factors acting in concert is important because they can display the intrinsic characters in soil microbial ecology. To address the effects of multiple-factors on soil bacteria, partial Mantel test and PLS-PM have been used in our study. Altitudinal gradients had the significant effects on soil bacteria community compositions, supported by the fact that, altitude gradients associating with abiotic changes, including temperature and precipitation, can build soil bacterial communities in their corresponding habitat (Sundqvist et al., 2013). Besides that, soil physical changes along altitudinal gradients had directly adverse effects on microbial community compositions and diversities, and the soil chemical properties in their corresponding habitat were favorable to the appearing of specific species relating to the microbial community diversity changes (Ogola et al., 2021; Anselmo and Rizzioli, 2022). In this respect, soil physical changes had negative effects on microbial community compositions and diversities, and soil chemical property changes had positive effects on microbial community diversities. However, soil physical and chemical properties usually change with depths so that different soil layer harbors distinct microbial communities and the response of bacterial community to environmental changes depends on soil depth (Barbour et al., 2022). Consequently, the responses of microbial community compositions to soil chemical property changes were different, and altitudinal gradients had different effects on soil microbial community compositions and diversities in all layers. In spite of this, there still has some knowledge gaps existed on the responses of bacteria to altitudinal gradients. Then, future work should be focusing on their evolutionary and physiological processes in response to elevation gradient changes that could usefully move the understanding of soil microbial ecology forward.

## Conclusion

Our results showed that soil microbial community compositions varied along altitudinal gradient in Yunnan karst graben basin due to environmental filtering. Because unique/endemic species restricted in specific habitats could become dominant under proper conditions and are important contributors to soil microbial diversities, high Shannon indexes were found in low altitude areas with changing environment conditions. The monotonically decreased Shannon indexes with elevation increasing in Mount CMD and Mount WGS also demonstrated that the coordinated patterns of Shannon indexes may be monotonically decreased along altitudinal gradients. Considering that unique species are not enormous in high altitude areas, the low proportions of unique OTUs appear at high altitude areas, which conforms to the changed regularities of Shannon indexes and impacts the widely accepted elevation Rapoport’s rules. Moreover, edaphic parameters were different in all layers, and SOC and TN increased monotonically with elevation increasing. The dominant *Bradyrhizobium* (alphaproteobacterial OTU 1) identified at high altitudes in all layers constitutes the important group of free-living diazotrophs and could bring fixed N into soils, which simultaneously enhances SOC and TN accumulation at high altitudes. Due to the responses of bacterial community to environmental changes varying with soil depths, the altitudinal gradients had different effects on soil microbial community compositions and diversities in all layers. Though it is the primary work about soil microbial community structure and diversity varying along altitudinal gradients in karst graben basin of Yunnan-Kweichow Plateau, our finding provides powerful information that can improve our better understanding of soil microbial ecology along altitudinal gradients at karst areas.

## Data availability statement

The datasets presented in this study can be found in online repositories. The names of the repository/repositories and accession number(s) can be found in the article/Supplementary material.

## Author contributions

QL designed the study and wrote the paper. QL, YL, and GL collected the soil samples. QL and JQ conducted the experiments and data analysis. All authors critically commented on and contributed to the manuscript.

## Funding

This study was supported by the National Key Research and Development Program of China (2016YFC0502501) and the Key Research and Development Program of Guangxi (GuikeAD20297091).

## Conflict of interest

The authors declare that they have no known competing financial interests or personal relationships that could have appeared to influence the work reported in this paper.

## Publisher’s note

All claims expressed in this article are solely those of the authors and do not necessarily represent those of their affiliated organizations, or those of the publisher, the editors and the reviewers. Any product that may be evaluated in this article, or claim that may be made by its manufacturer, is not guaranteed or endorsed by the publisher.

## References

[ref1] Amin M. M. Akter A. Jahangir M. M. R. Ahmed T. (2021). Leaching and runoff potential of nutrient and water losses in rice field as affected by alternate wetting and drying irrigation. J. Environ. Manag. 297:113402. doi: 10.1016/j.jenvman.2021.113402, PMID: 34333312

[ref2] Anderson M. J. Walsh D. C. (2013). PERMANOVA, ANOSIM, and the mantel test in the face of heterogeneous dispersions: what null hypothesis are you testing? Ecol. Monogr. 83, 557–574. doi: 10.1890/12-2010.1

[ref3] Anselmo L. Rizzioli B. (2022). The small range and the great threat: extinction risk assessment of the narrow endemism *Carabus cychroides* under climate change. J. Insect Conserv. 26, 17–27. doi: 10.1007/s10841-021-00357-0

[ref4] Baldrian P. (2019). The known and the unknown in soil microbial ecology. FEMS Microbiol. Ecol. 95:fiz005. doi: 10.1093/femsec/fiz005 30624643

[ref5] Banerjee S. Schlaeppi K. van der Heijden M. G. (2018). Keystone taxa as drivers of microbiome structure and functioning. Nat. Rev. Microbiol. 16, 567–576. doi: 10.1038/s41579-018-0024-1, PMID: 29789680

[ref6] Barbour K. M. Weihe C. Allison S. D. Martiny J. B. (2022). Bacterial community response to environmental change varies with depth in the surface soil. Soil Biol. Biochem. 172:108761. doi: 10.1016/j.soilbio.2022.108761

[ref7] Baveye P. C. Schnee L. S. Boivin P. Laba M. Radulovich R. (2020). Soil organic matter research and climate change: merely re-storing carbon versus restoring soil functions. Front. Env. Sci. 8:579904. doi: 10.3389/fenvs.2020.579904

[ref8] Bhat J. A. Kumar M. Negi A. K. Todaria N. P. Malik Z. A. Pala N. A. (2020). Species diversity of woody vegetation along altitudinal gradient of the Western Himalayas. Glob. Ecol. Conserv. 24:e01302. doi: 10.1016/j.gecco.2020.e01302

[ref9] Brown J. H. (2001). Mammals on mountainsides: Elevational patterns of diversity. Glob. Ecol. Biogeogr. 10, 101–109. doi: 10.1046/j.1466-822x.2001.00228.x

[ref10] Bryant J. A. Lamanna C. Morlon H. Kerkhoff A. J. Enquist B. J. Green J. L. (2008). Microbes on mountainsides: contrasting elevational patterns of bacterial and plant diversity. Proc. Natl. Acad. Sci. U.S.A. 105, 11505–11511. doi: 10.1073/pnas.0801920105, PMID: 18695215 PMC2556412

[ref11] Cai Z. Wang X. Bhadra S. Gao Q. (2020). Distinct factors drive the assembly of quinoa-associated microbiomes along elevation. Plant Soil 448, 55–69. doi: 10.1007/s11104-019-04387-1

[ref12] Dai Z. Zang H. Chen J. Fu Y. Wang X. Liu H. (2021). Metagenomic insights into soil microbial communities involved in carbon cycling along an elevation climosequences. Environ. Microbiol. 23, 4631–4645. doi: 10.1111/1462-2920.15655, PMID: 34190385

[ref13] Delgado-Baquerizo M. Oliverio A. M. Brewer T. E. Benavent-González A. Eldridge D. J. Bardgett R. D. (2018). A global atlas of the dominant bacteria found in soil. Science 359, 320–325. doi: 10.1126/science.aap9516, PMID: 29348236

[ref14] Fierer N. McCain C. M. Meir P. Zimmermann M. Rapp J. M. Silman M. R. (2011). Microbes do not follow the elevational diversity patterns of plants and animals. Ecology 92, 797–804. doi: 10.1890/10-1170.1 21661542

[ref15] Fitzgerald D. M. Rosenberg S. M. (2019). What is mutation? A chapter in the series: how microbes “jeopardize” the modern synthesis. PLoS Genet. 15:e1007995. doi: 10.1371/journal.pgen.1007995, PMID: 30933985 PMC6443146

[ref16] Gao D. Bai E. Li M. Zhao C. Yu K. Hagedorn F. (2020). Responses of soil nitrogen and phosphorus cycling to drying and rewetting cycles: a meta-analysis. Soil Biol. Biochem. 148:107896. doi: 10.1016/j.soilbio.2020.107896

[ref17] Grau O. Grytnes J. A. Birks H. J. B. (2007). A comparison of altitudinal species richness patterns of bryophytes with other plant groups in Nepal, Central Himalaya. J. Biogeogr. 34, 1907–1915. doi: 10.1111/j.1365-2699.2007.01745.x

[ref18] Guo Q. Kelt D. A. Sun Z. Liu H. Hu L. Ren H. (2013). Global variation in elevational diversity patterns. Sci. Rep. 3:3007. doi: 10.1038/srep03007, PMID: 24157658 PMC6505670

[ref19] Hayat W. Khan S. Hayat M. T. Pervez R. Ahmad S. Iqbal A. (2021). The effect of deforestation on soil quality in lesser-Himalayan community forests of Abbottabad, Pakistan. Arab. J. Geosci. 14, 1–14. doi: 10.1007/s12517-021-08271-0

[ref20] Hu L. Li Q. Yan J. Liu C. Zhong J. (2022). Vegetation restoration facilitates belowground microbial network complexity and recalcitrant soil organic carbon storage in Southwest China karst region. Sci. Total Environ. 820:153137. doi: 10.1016/j.scitotenv.2022.153137, PMID: 35041964

[ref21] Jiao S. Lu Y. (2020). Soil pH and temperature regulate assembly processes of abundant and rare bacterial communities in agricultural ecosystems. Environ. Microbiol. 22, 1052–1065. doi: 10.1111/1462-2920.14815, PMID: 31599105

[ref22] Jobbágy E. G. Jackson R. B. (2001). The distribution of soil nutrients with depth: global patterns and the imprint of plants. Biogeochemistry 53, 51–77. doi: 10.1023/A:1010760720215

[ref23] Kardol P. Cregger M. A. Campany C. E. Classen A. T. (2010). Soil ecosystem functioning under climate change: plant species and community effects. Ecology 91, 767–781. doi: 10.1890/09-0135.1 20426335

[ref24] Karimi B. Terrat S. Dequiedt S. Saby N. P. A. Horrigue W. Lelièvre M. (2018). Biogeography of soil bacteria and archaea across France. Sci. Adv. 4:eaat1808. doi: 10.1126/sciadv.aat1808 29978046 PMC6031370

[ref25] Keller S. (2022). Vegetation and soil microbial diversity along alpine elevation and snow gradients [dissertation/master’s thesis]. [Winterthur(IL)]: ZHAW Zürcher Hochschule für Angewandte Wissenschaften. doi: 10.21256/zhaw-25202

[ref26] Körner C. (2007). The use of ‘altitude’ in ecological research. Trends Ecol. Evol. 22, 569–574. doi: 10.1016/j.tree.2007.09.006 17988759

[ref27] Kumar S. Suyal D. C. Yadav A. Shouche Y. Goel R. (2019). Microbial diversity and soil physiochemical characteristic of higher altitude. PLoS One 14:e0213844. doi: 10.1371/journal.pone.0213844, PMID: 30875404 PMC6419999

[ref28] Langenheder S. Lindström E. S. (2019). Factors influencing aquatic and terrestrial bacterial community assembly. Environ. Microbiol. Rep. 11, 306–315. doi: 10.1111/1758-2229.12731, PMID: 30618071

[ref29] Leeming E. R. Johnson A. J. Spector T. D. Le Roy C. I. (2019). Effect of diet on the gut microbiota: rethinking intervention duration. Nutrients 11:2862. doi: 10.3390/nu11122862, PMID: 31766592 PMC6950569

[ref30] Lennon J. T. Jones S. E. (2011). Microbial seed banks: the ecological and evolutionary implications of dormancy. Nat. Rev. Microbiol. 9, 119–130. doi: 10.1038/nrmicro2504, PMID: 21233850

[ref31] Li L. Zhang Y. Wu J. Li S. Zhang B. Zu J. (2019). Increasing sensitivity of alpine grasslands to climate variability along an elevational gradient on the Qinghai-Tibet Plateau. Sci. Total Environ. 678, 21–29. doi: 10.1016/j.scitotenv.2019.04.399, PMID: 31075588

[ref32] Liang C. Schimel J. P. Jastrow J. D. (2017). The importance of anabolism in microbial control over soil carbon storage. Nat. Microbiol. 2:17105. doi: 10.1038/nmicrobiol.2017.105 28741607

[ref33] Looby C. I. Martin P. H. (2020). Diversity and function of soil microbes on montane gradients: the state of knowledge in a changing world. FEMS Microbiol. Ecol. 96:fiaa122. doi: 10.1093/femsec/fiaa122, PMID: 32780840

[ref34] Lynch M. D. Neufeld J. D. (2015). Ecology and exploration of the rare biosphere. Nat. Rev. Microbiol. 13, 217–229. doi: 10.1038/nrmicro3400, PMID: 25730701

[ref35] Mirmohamadsadeghi S. Karimi K. Azarbaijani R. Yeganeh L. P. Angelidaki I. Nizami A. S. (2021). Pretreatment of lignocelluloses for enhanced biogas production: a review on influencing mechanisms and the importance of microbial diversity. Renew. Sust. Energ. Rev. 135:110173. doi: 10.1016/j.rser.2020.110173

[ref36] Nottingham A. T. Fierer N. Turner B. L. Whitaker J. Ostle N. J. McNamara N. P. (2018). Microbes follow Humboldt: temperature drives plant and soil microbial diversity patterns from the Amazon to the Andes. Ecology 99, 2455–2466. doi: 10.1002/ecy.2482, PMID: 30076592 PMC6850070

[ref37] Ogola H. J. O. Selvarajan R. Tekere M. (2021). Local geomorphological gradients and land use patterns play key role on the soil bacterial community diversity and dynamics in the highly endemic indigenous afrotemperate coastal scarp forest biome. Front. Microbiol. 12:592725. doi: 10.3389/fmicb.2021.592725, PMID: 33716998 PMC7943610

[ref38] Pham T. G. Nguyen H. T. Kappas M. (2018). Assessment of soil quality indicators under different agricultural land uses and topographic aspects in Central Vietnam. Int. Soil Water Conse. 6, 280–288. doi: 10.1016/j.iswcr.2018.08.001

[ref39] Phillips J. D. (2016). Landforms as extended composite phenotypes. Earth Surf. Process. Landf. 41, 16–26. doi: 10.1002/esp.3764

[ref40] Qiu J. Cao J. Lan G. Liang Y. Wang H. Li Q. (2020). The influence of land use patterns on soil bacterial community structure in the karst graben basin of Yunnan province, China. Forests 11:51. doi: 10.3390/f11010051

[ref41] Rillig M. C. Ryo M. Lehmann A. Aguilar-Trigueros C. A. Buchert S. Wulf A. (2019). The role of multiple global change factors in driving soil functions and microbial biodiversity. Science 366, 886–890. doi: 10.1126/science.aay2832, PMID: 31727838 PMC6941939

[ref42] Shen C. Shi Y. Fan K. He J. S. Adams J. M. Ge Y. (2019). Soil pH dominates elevational diversity pattern for bacteria in high elevation alkaline soils on the Tibetan Plateau. FEMS Microbiol. Ecol. 95:fiz003. doi: 10.1093/femsec/fiz003 30629166

[ref43] Shimuta T. R. Nakano K. Yamaguchi Y. Ozaki S. Fujimitsu K. Matsunaga C. (2004). Novel heat shock protein *HspQ* stimulates the degradation of mutant DnaA protein in Escherichia coli. Genes Cells 9, 1151–1166. doi: 10.1111/j.1365-2443.2004.00800.x, PMID: 15569148

[ref44] Simpson G. G. (1980). Splendid isolation: The curious history of south American mammals Yale Univ. Press.

[ref45] Singh D. Takahashi K. Kim M. Chun J. Adams J. M. (2012). A hump-backed trend in bacterial diversity with elevation on Mount Fuji, Japan. Microb. Ecol. 63, 429–437. doi: 10.1007/s00248-011-9900-1, PMID: 21735154

[ref46] Stevens G. C. (1992). The elevational gradient in altitudinal range: an extension of Rapoport's latitudinal rule to altitude. Am. Nat. 140, 893–911. doi: 10.1086/285447, PMID: 19426029

[ref47] Sundqvist M. K. Sanders N. J. Wardle D. A. (2013). Community and ecosystem responses to elevational gradients: processes, mechanisms, and insights for global change. Annu. Rev. Ecol. Evol. Syst. 44, 261–280. doi: 10.1146/annurev-ecolsys-110512-135750

[ref48] Tao J. Wang S. Liao T. Luo H. (2021). Evolutionary origin and ecological implication of a unique nif island in free-living Bradyrhizobium lineages. ISME J. 15, 3195–3206. doi: 10.1038/s41396-021-01002-z, PMID: 33990706 PMC8528876

[ref49] Van Der Heijden M. G. Bardgett R. D. Van Straalen N. M. (2008). The unseen majority: soil microbes as drivers of plant diversity and productivity in terrestrial ecosystems. Ecol. Lett. 11, 296–310. doi: 10.1111/j.1461-0248.2007.01139.x, PMID: 18047587

[ref50] Vetaas O. R. Grytnes J. A. (2002). Distribution of vascular plant species richness and endemic richness along the Himalayan elevation gradient in Nepal. Glob. Ecol. Biogeogr. 11, 291–301. doi: 10.1046/j.1466-822X.2002.00297.x

[ref51] Wang J. Gong B. Wang Y. Wen Y. Zhou J. He Q. (2017). The potential multiple mechanisms and microbial communities in simultaneous nitrification and denitrification process treating high carbon and nitrogen concentration saline wastewater. Bioresour. Technol. 243, 708–715. doi: 10.1016/j.biortech.2017.06.131, PMID: 28710998

[ref52] Wang Y. Zhang H. Zhang G. Wang B. Peng S. H. He R. S. (2017). Zoning of environmental geology and functions in karst fault-depression basins. Carsol. Sin. 36, 283–295. (In Chinese with English abstract) doi: 10.11932/karst20170316

[ref53] Wieder W. R. Pierson D. Earl S. Lajtha K. Baer S. G. Ballantyne F. (2021). SoDaH: the SOils DAta harmonization database, an open-source synthesis of soil data from research networks, version 1.0. Earth Syst. Sci. Data 13, 1843–1854. doi: 10.5194/essd-13-1843-2021

[ref54] Xun W. Huang T. Zhao J. Ran W. Wang B. Shen Q. (2015). Environmental conditions rather than microbial inoculum composition determine the bacterial composition, microbial biomass and enzymatic activity of reconstructed soil microbial communities. Soil Biol. Biochem. 90, 10–18. doi: 10.1016/j.soilbio.2015.07.018

[ref55] Yan J. Li Q. Hu L. Wang J. Zhou Q. Zhong J. (2022). Response of microbial communities and their metabolic functions to calcareous succession process. Sci. Total Environ. 825:154020. doi: 10.1016/j.scitotenv.2022.154020, PMID: 35202682

[ref56] Yin Z. Shan Z. J. Qin W. Yu Y. Guo Q. K. Li B. (2020). Preliminary research on a method of outcrops extraction on karst gabin ecosystem based on digital image processing: the case of the Mengzi Gabin Basin. Forest. Environ. Sci. 36, 26–33. (In Chinese with English abstract) doi: 10.3969/j.issn.1006-4427.2020.06.005

[ref57] Yuan D. X. (2001). On the karst ecosystem. Acta Geol. Sin. 75, 336–338. doi: 10.1111/j.1755-6724.2001.tb00541.x

[ref58] Zhou Y. Ochola A. C. Njogu A. W. Boru B. H. Mwachala G. Hu G. (2019). The species richness pattern of vascular plants along a tropical elevational gradient and the test of elevational Rapoport's rule depend on different life-forms and phytogeographic affinities. Ecol. Evol. 9, 4495–4503. doi: 10.1002/ece3.5027, PMID: 31031922 PMC6476750

