# Supplementary material for: Soil bacterial community changes along elevation gradients in karst graben basin of Yunnan-Kweichow Plateau
Source: Front Microbiol. 2022 Dec 22;13:1054667. doi: 10.3389/fmicb.2022.1054667 (PMC9813600; doi:10.3389/fmicb.2022.1054667)
Supplement: Supplementary file 5 [file Data_Sheet_1.docx]

Soil bacterial community changes along elevation gradients in karst graben basin of Yunnan-Kweichow Plateau

Qiang Li^1,2*^, Jiangmei Qiu^1,2^, Yueming Liang^1,2^, Gaoyong Lan^1,2^

^1^Key Laboratory of Karst Ecosystem and Treatment of Rocky Desertification, MNR, Key Laboratory of Karst Dynamics, MNR & GZAR, Institute of Karst Geology, Chinese Academy of Geological Sciences, Guilin 541004, PR China

^2^International Research Center on Karst under the Auspices of UNESCO, Guilin 541004, China

^*^Correspondence: Qiang Li E-mail: [glqiangli@hotmail.com](mailto:glqiangli@hotmail.com)


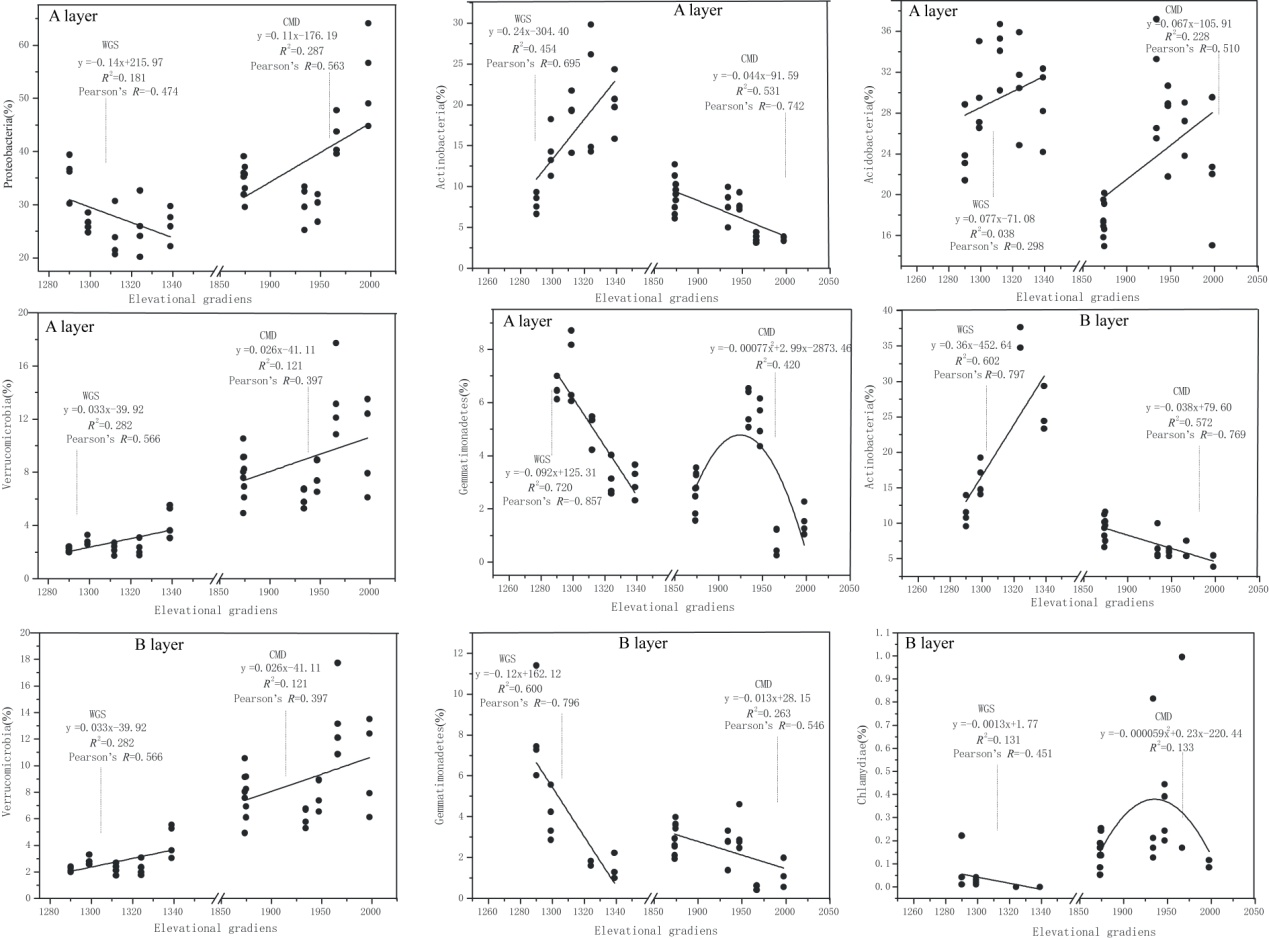


Figure S1 | The patterns of the top five phyla along altitudinal gradients.


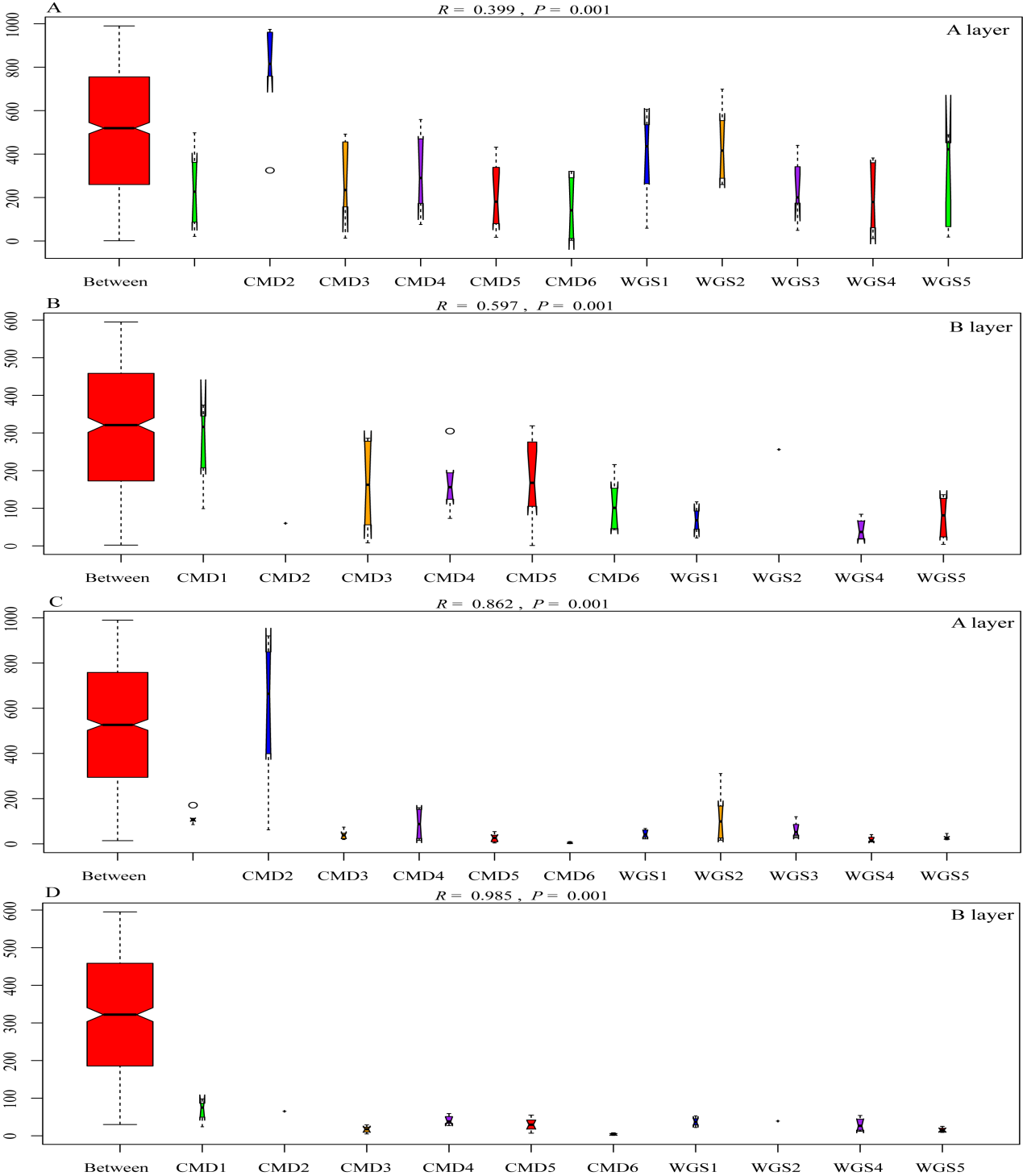


Figure S2 | ANOSIM analysis based on alpha diversities (A and B) and Bray-Curtis distances (C and D) representing the soil bacterial community similarity/dissimilarity from A layer and B layer.

Ordinate–the rank of the distance between samples; Abscissa–Between is the result along altitudinal gradients, and the others are the results within their groups, respectively. R = 1 signifies differences between groups, while R = 0 signifies that the groups are identical.


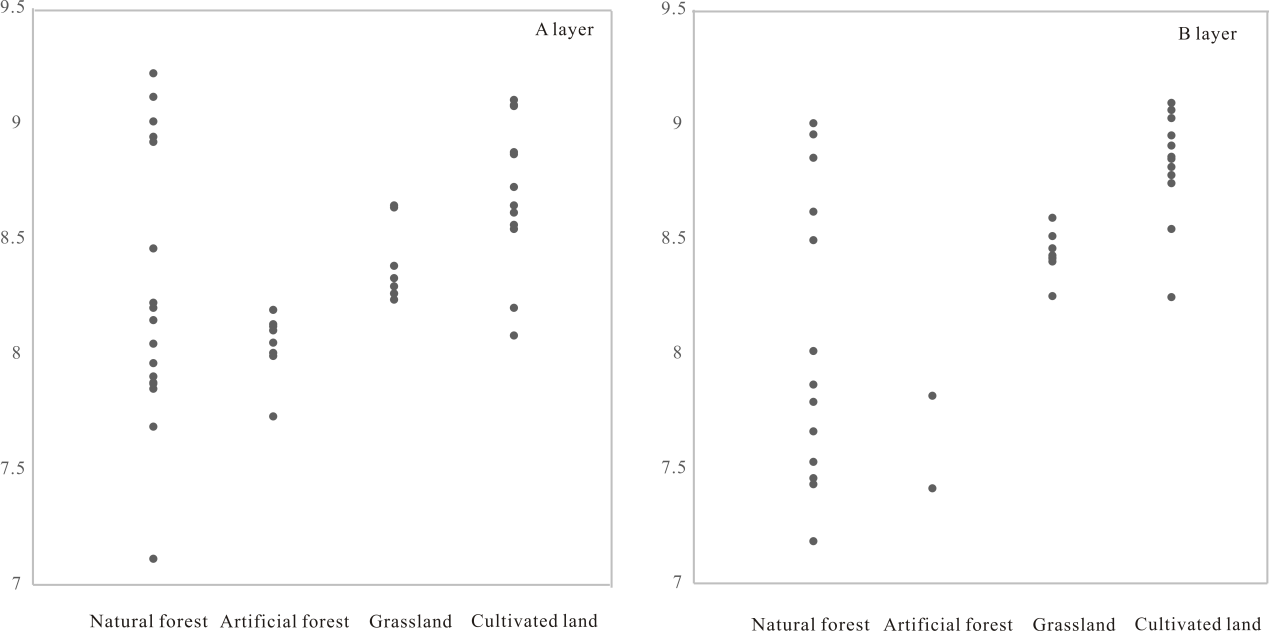


Figure S3 | Shannon indexes with vegetation type variations along altitudinal gradients.
